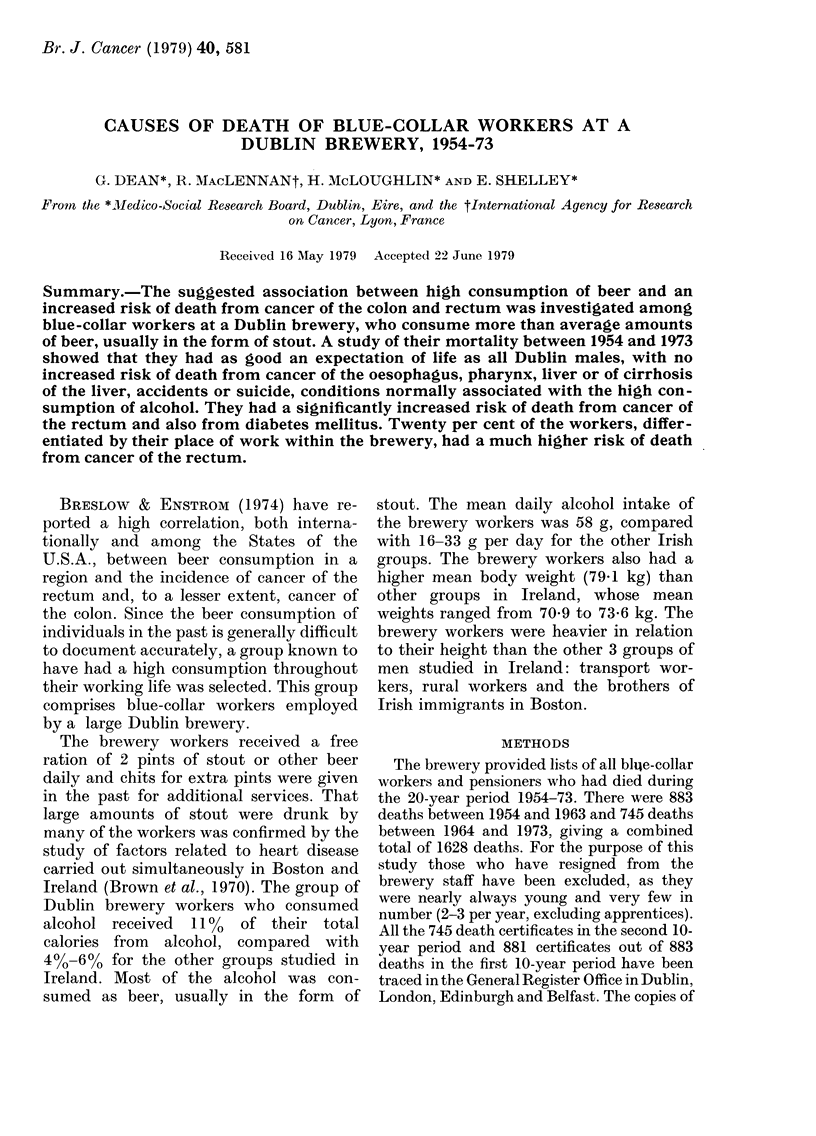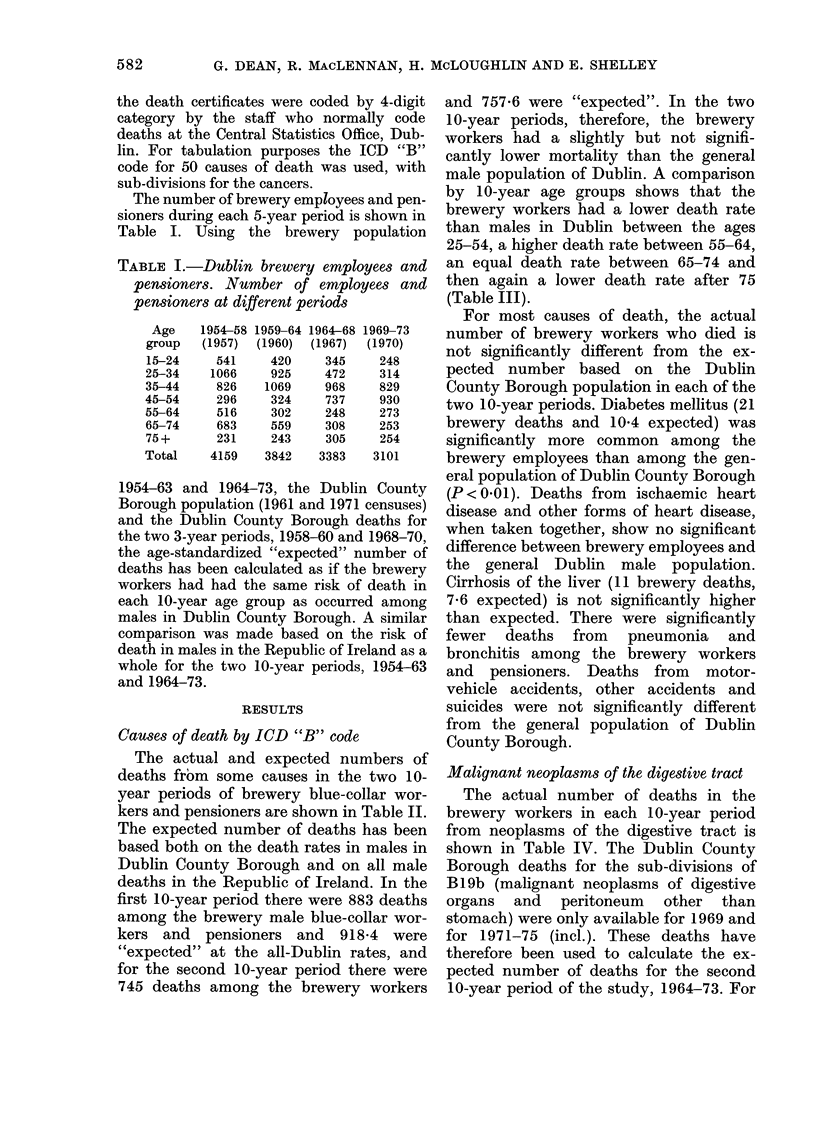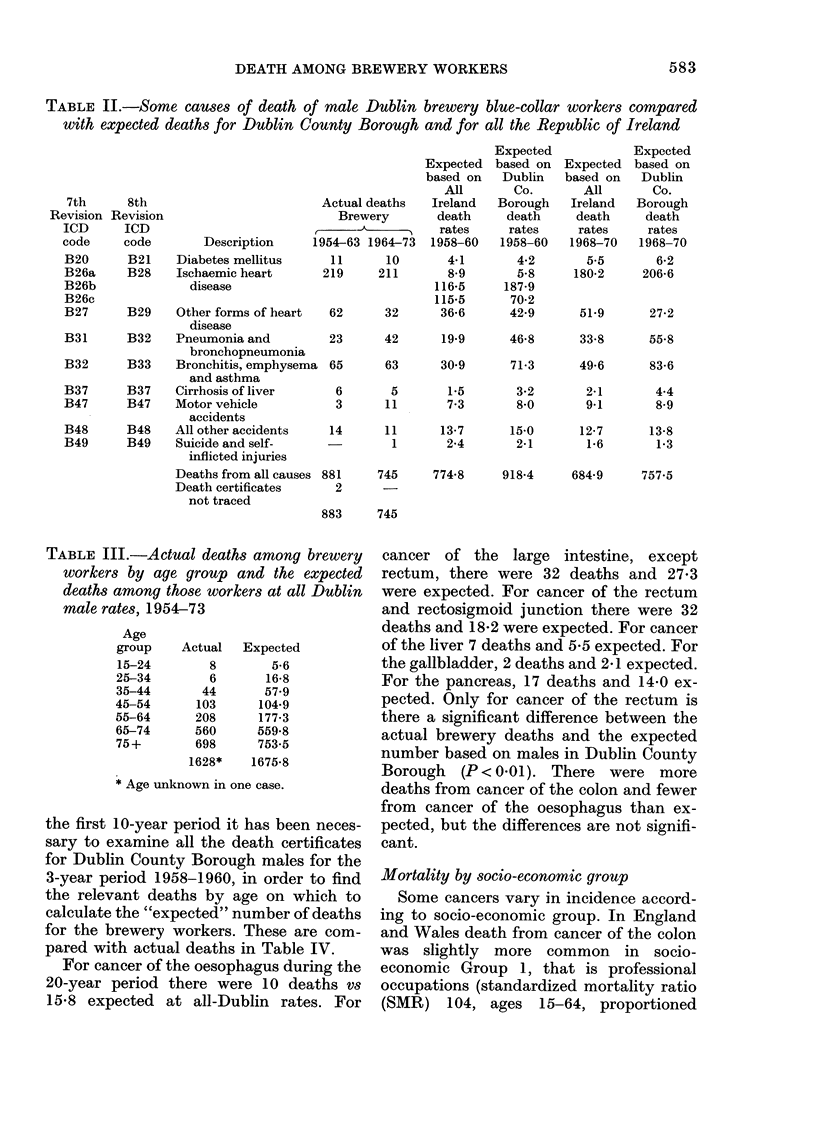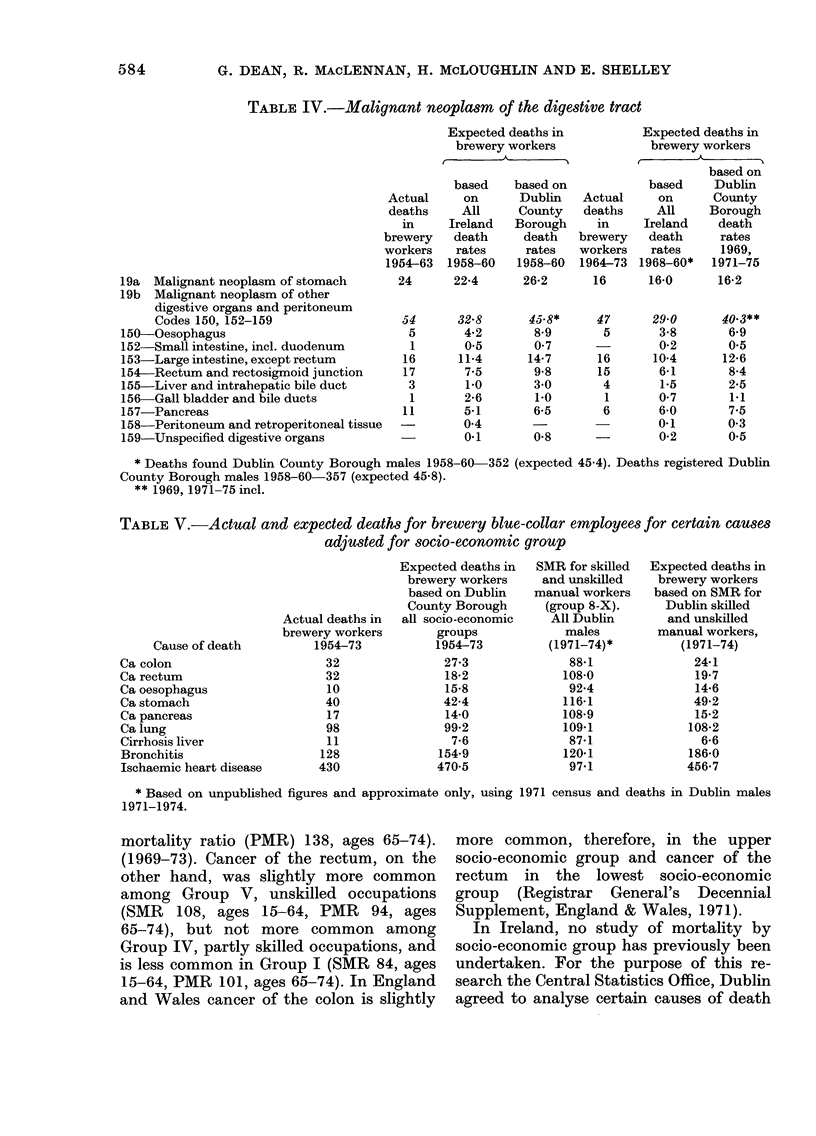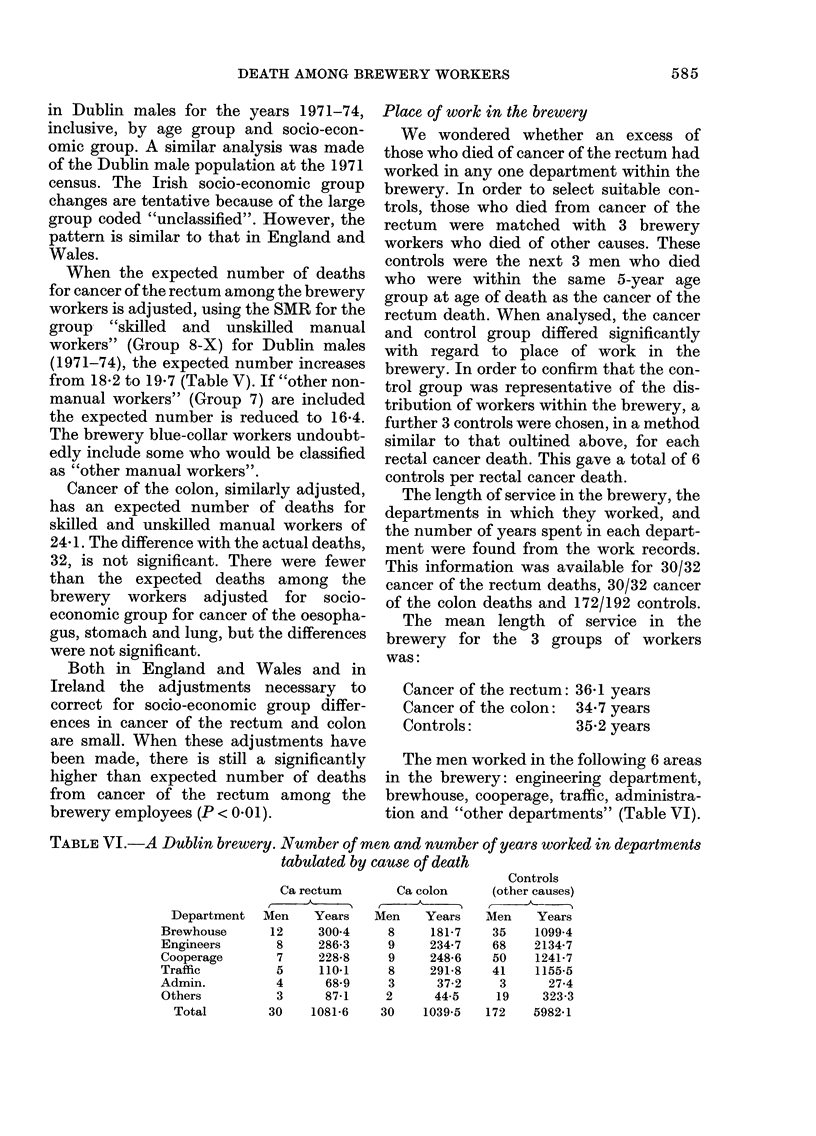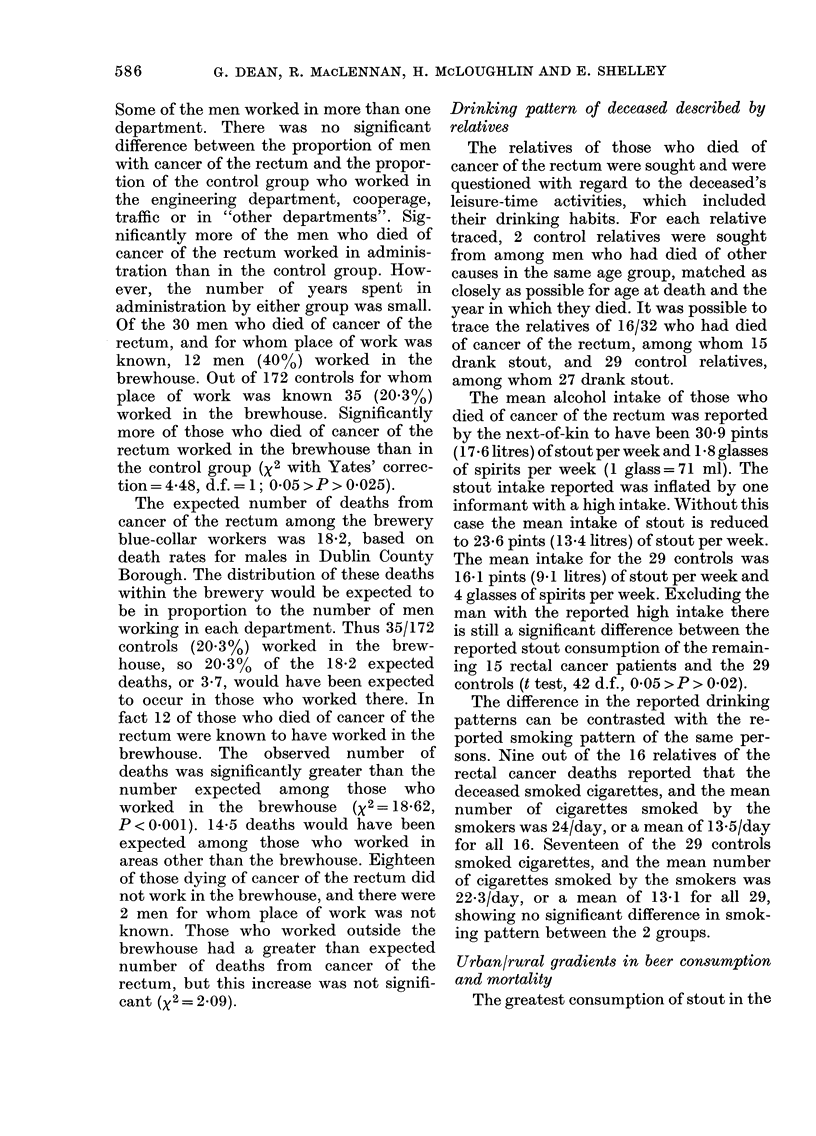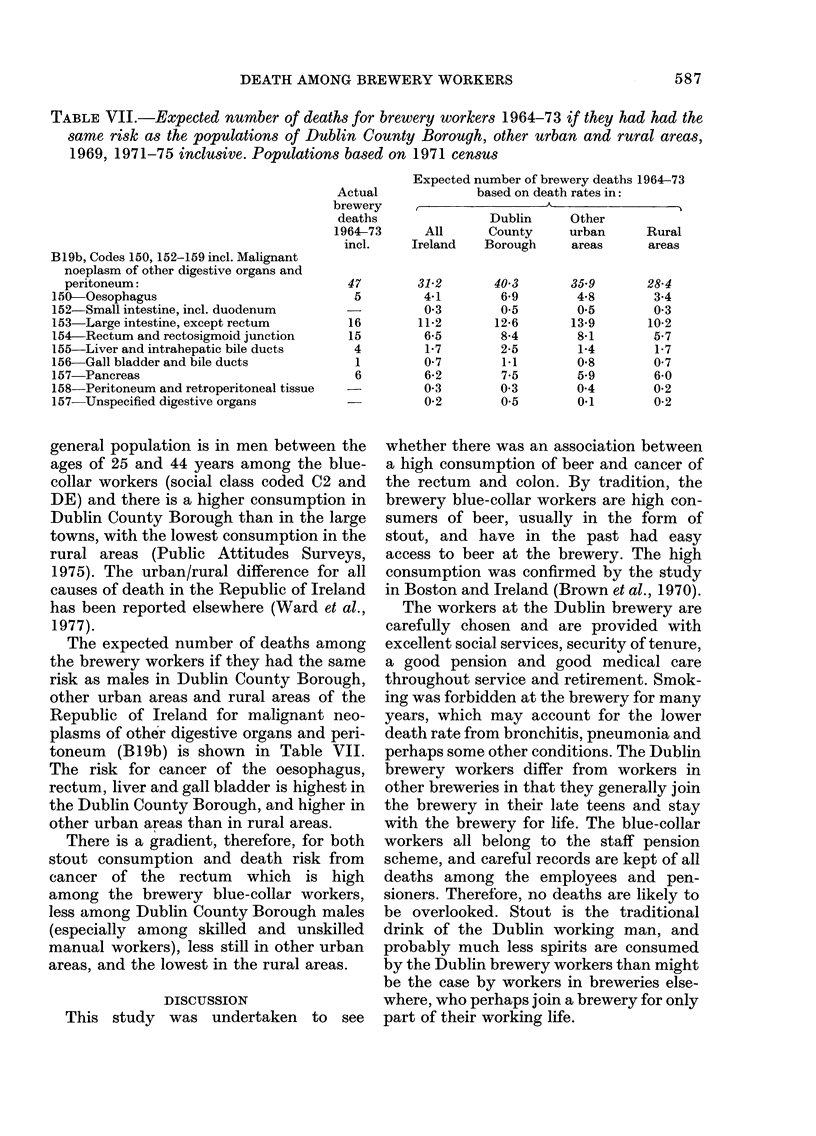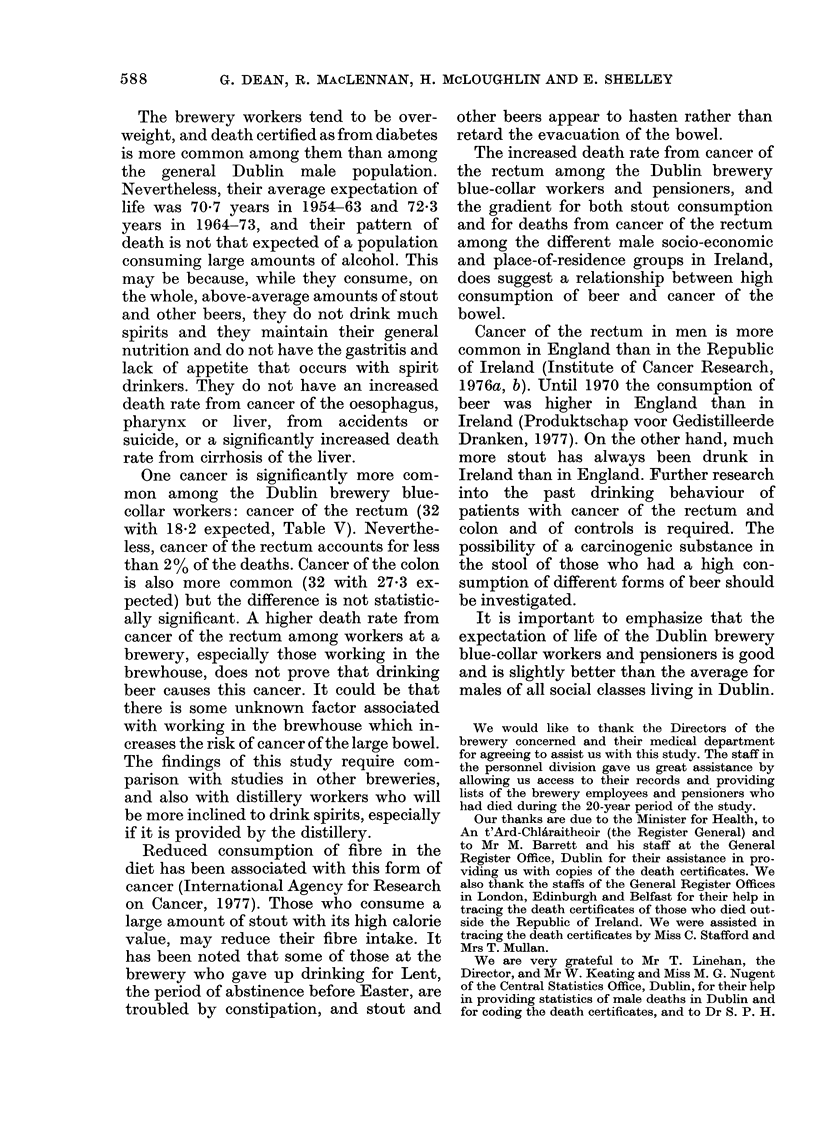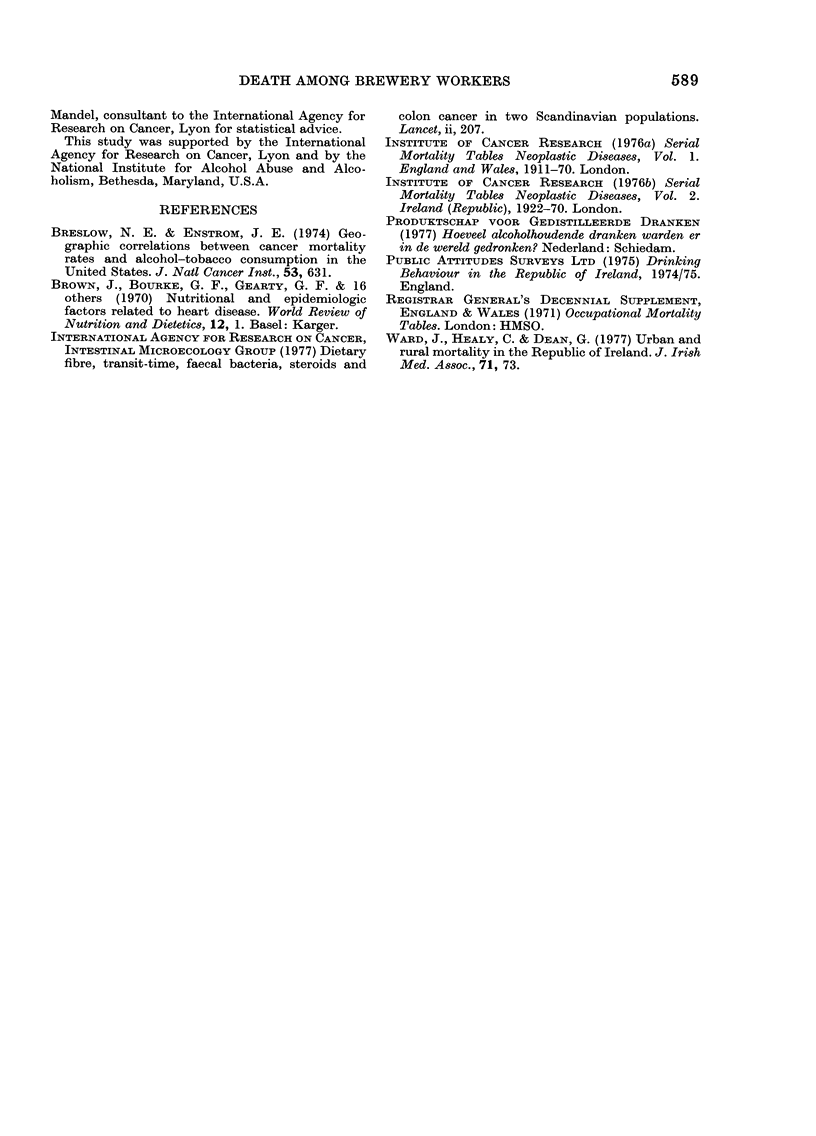# Causes of death of blue-collar workers at a Dublin brewery, 1954--73.

**DOI:** 10.1038/bjc.1979.223

**Published:** 1979-10

**Authors:** G. Dean, R. MacLennan, H. McLoughlin, E. Shelley

## Abstract

The suggested association between high consumption of beer and an increased risk of death from cancer of the colon and rectum was investigated among blue-collar workers at a Dublin brewery, who consume more than average amounts of beer, usually in the form of stout. A study of their mortality between 1954 and 1973 showed that they had as good an expectation of life as all Dublin males, with no increased risk of death from cancer of the oesophagus, pharynx, liver or of cirrhosis of the liver, accidents or suicide, conditions normally associated with the high consumption of alcohol. They had significantly increased risk of death from cancer of the rectum and also from diabetes mellitus. Twenty per cent of the workers, differentiated by their place of work within the brewery, had a much higher risk of death from cancer of the rectum.


*Br. J. Cancer* (1979) **40**, 581

### CAUSES OF DEATH OF BLUE-COLLAR WORKERS AT A DUBLIN BREWERY, 1954-73

G. DEAN*, R. MacLENNAN†, H. McLOUGHLIN* and E. SHELLEY*

*From the *Medico-Social Research Board, Dublin, Eire, and the †International Agency for Research on Cancer, Lyon, France*

Received 16 May 1979 Accepted 22 June 1979

**Summary.—The suggested association between high consumption of beer and an increased risk of death from cancer of the colon and rectum was investigated among blue-collar workers at a Dublin brewery, who consume more than average amounts of beer, usually in the form of stout. A study of their mortality between 1954 and 1973 showed that they had as good an expectation of life as all Dublin males, with no increased risk of death from cancer of the oesophagus, pharynx, liver or of cirrhosis of the liver, accidents or suicide, conditions normally associated with the high consumption of alcohol. They had a significantly increased risk of death from cancer of the rectum and also from diabetes mellitus. Twenty per cent of the workers, differentiated by their place of work within the brewery, had a much higher risk of death from cancer of the rectum.**

Breslow & Enstrom (1974) have reported a high correlation, both internationally and among the States of the U.S.A., between beer consumption in a region and the incidence of cancer of the rectum and, to a lesser extent, cancer of the colon. Since the beer consumption of individuals in the past is generally difficult to document accurately, a group known to have had a high consumption throughout their working life was selected. This group comprises blue-collar workers employed by a large Dublin brewery.

The brewery workers received a free ration of 2 pints of stout or other beer daily and chits for extra pints were given in the past for additional services. That large amounts of stout were drunk by many of the workers was confirmed by the study of factors related to heart disease carried out simultaneously in Boston and Ireland (Brown *et al.*, 1970). The group of Dublin brewery workers who consumed alcohol received 11% of their total calories from alcohol, compared with 4%–6% for the other groups studied in Ireland. Most of the alcohol was consumed as beer, usually in the form of stout. The mean daily alcohol intake of the brewery workers was 58 g, compared with 16–33 g per day for the other Irish groups. The brewery workers also had a higher mean body weight (79·1 kg) than other groups in Ireland, whose mean weights ranged from 70·9 to 73·6 kg. The brewery workers were heavier in relation to their height than the other 3 groups of men studied in Ireland: transport workers, rural workers and the brothers of Irish immigrants in Boston.

### METHODS

The brewery provided lists of all blue-collar workers and pensioners who had died during the 20-year period 1954–73. There were 883 deaths between 1954 and 1963 and 745 deaths between 1964 and 1973, giving a combined total of 1628 deaths. For the purpose of this study those who have resigned from the brewery staff have been excluded, as they were nearly always young and very few in number (2–3 per year, excluding apprentices). All the 745 death certificates in the second 10-year period and 881 certificates out of 883 deaths in the first 10-year period have been traced in the General Register Office in Dublin, London, Edinburgh and Belfast. The copies of the death certificates were coded by 4-digit category by the staff who normally code deaths at the Central Statistics Office, Dublin. For tabulation purposes the ICD "B" code for 50 causes of death was used, with sub-divisions for the cancers.

The number of brewery employees and pensioners during each 5-year period is shown in Table I. Using the brewery population

TABLE I.—*Dublin brewery employees and pensioners. Number of employees and pensioners at different periods*

| Age group | 1954–58 (1957) | 1959–64 (1960) | 1964–68 (1967) | 1969–73 (1970) |
|---|---|---|---|---|
| 15–24 | 541 | 420 | 345 | 248 |
| 25–34 | 1066 | 925 | 472 | 314 |
| 35–44 | 826 | 1069 | 968 | 829 |
| 45–54 | 296 | 324 | 737 | 930 |
| 55–64 | 516 | 302 | 248 | 273 |
| 65–74 | 683 | 559 | 308 | 253 |
| 75+ | 231 | 243 | 305 | 254 |
| Total | 4159 | 3842 | 3383 | 3101 |

1954–63 and 1964–73, the Dublin County Borough population (1961 and 1971 censuses) and the Dublin County Borough deaths for the two 3-year periods, 1958–60 and 1968–70, the age-standardized "expected" number of deaths has been calculated as if the brewery workers had had the same risk of death in each 10-year age group as occurred among males in Dublin County Borough. A similar comparison was made based on the risk of death in males in the Republic of Ireland as a whole for the two 10-year periods, 1954–63 and 1964–73.

### RESULTS

### *Causes of death by ICD "B" code*

The actual and expected numbers of deaths from some causes in the two 10-year periods of brewery blue-collar workers and pensioners are shown in Table II. The expected number of deaths has been based both on the death rates in males in Dublin County Borough and on all male deaths in the Republic of Ireland. In the first 10-year period there were 883 deaths among the brewery male blue-collar workers and pensioners and 918·4 were "expected" at the all-Dublin rates, and for the second 10-year period there were 745 deaths among the brewery workers and 757·6 were "expected". In the two 10-year periods, therefore, the brewery workers had a slightly but not significantly lower mortality than the general male population of Dublin. A comparison by 10-year age groups shows that the brewery workers had a lower death rate than males in Dublin between the ages 25–54, a higher death rate between 55–64, an equal death rate between 65–74 and then again a lower death rate after 75 (Table III).

For most causes of death, the actual number of brewery workers who died is not significantly different from the expected number based on the Dublin County Borough population in each of the two 10-year periods. Diabetes mellitus (21 brewery deaths and 10·4 expected) was significantly more common among the brewery employees than among the general population of Dublin County Borough ($P < 0{\cdot}01$). Deaths from ischaemic heart disease and other forms of heart disease, when taken together, show no significant difference between brewery employees and the general Dublin male population. Cirrhosis of the liver (11 brewery deaths, 7·6 expected) is not significantly higher than expected. There were significantly fewer deaths from pneumonia and bronchitis among the brewery workers and pensioners. Deaths from motor-vehicle accidents, other accidents and suicides were not significantly different from the general population of Dublin County Borough.

### *Malignant neoplasms of the digestive tract*

The actual number of deaths in the brewery workers in each 10-year period from neoplasms of the digestive tract is shown in Table IV. The Dublin County Borough deaths for the sub-divisions of B19b (malignant neoplasms of digestive organs and peritoneum other than stomach) were only available for 1969 and for 1971–75 (incl.). These deaths have therefore been used to calculate the expected number of deaths for the second 10-year period of the study, 1964–73. For

TABLE II.—*Some causes of death of male Dublin brewery blue-collar workers compared with expected deaths for Dublin County Borough and for all the Republic of Ireland*

| 7th Revision ICD code | 8th Revision ICD code | Description | Actual deaths Brewery 1954–63 | Actual deaths Brewery 1964–73 | Expected based on All Ireland death rates 1958–60 | Expected based on Dublin Co. Borough death rates 1958–60 | Expected based on All Ireland death rates 1968–70 | Expected based on Dublin Co. Borough death rates 1968–70 |
|---|---|---|---|---|---|---|---|---|
| B20 | B21 | Diabetes mellitus | 11 | 10 | 4·1 | 4·2 | 5·5 | 6·2 |
| B26a | B28 | Ischaemic heart | 219 | 211 | 8·9 | 5·8 | 180·2 | 206·6 |
| B26b | | disease | | | 116·5 | 187·9 | | |
| B26c | | | | | 115·5 | 70·2 | | |
| B27 | B29 | Other forms of heart disease | 62 | 32 | 36·6 | 42·9 | 51·9 | 27·2 |
| B31 | B32 | Pneumonia and bronchopneumonia | 23 | 42 | 19·9 | 46·8 | 33·8 | 55·8 |
| B32 | B33 | Bronchitis, emphysema and asthma | 65 | 63 | 30·9 | 71·3 | 49·6 | 83·6 |
| B37 | B37 | Cirrhosis of liver | 6 | 5 | 1·5 | 3·2 | 2·1 | 4·4 |
| B47 | B47 | Motor vehicle accidents | 3 | 11 | 7·3 | 8·0 | 9·1 | 8·9 |
| B48 | B48 | All other accidents | 14 | 11 | 13·7 | 15·0 | 12·7 | 13·8 |
| B49 | B49 | Suicide and self-inflicted injuries | — | 1 | 2·4 | 2·1 | 1·6 | 1·3 |
| | | Deaths from all causes | 881 | 745 | 774·8 | 918·4 | 684·9 | 757·5 |
| | | Death certificates not traced | 2 | — | | | | |
| | | | 883 | 745 | | | | |

TABLE III.—*Actual deaths among brewery workers by age group and the expected deaths among those workers at all Dublin male rates*, 1954–73

| Age group | Actual | Expected |
|---|---|---|
| 15–24 | 8 | 5·6 |
| 25–34 | 6 | 16·8 |
| 35–44 | 44 | 57·9 |
| 45–54 | 103 | 104·9 |
| 55–64 | 208 | 177·3 |
| 65–74 | 560 | 559·8 |
| 75+ | 698 | 753·5 |
| | 1628* | 1675·8 |

* Age unknown in one case.

the first 10-year period it has been necessary to examine all the death certificates for Dublin County Borough males for the 3-year period 1958–1960, in order to find the relevant deaths by age on which to calculate the "expected" number of deaths for the brewery workers. These are compared with actual deaths in Table IV.

For cancer of the oesophagus during the 20-year period there were 10 deaths *vs* 15·8 expected at all-Dublin rates. For cancer of the large intestine, except rectum, there were 32 deaths and 27·3 were expected. For cancer of the rectum and rectosigmoid junction there were 32 deaths and 18·2 were expected. For cancer of the liver 7 deaths and 5·5 expected. For the gallbladder, 2 deaths and 2·1 expected. For the pancreas, 17 deaths and 14·0 expected. Only for cancer of the rectum is there a significant difference between the actual brewery deaths and the expected number based on males in Dublin County Borough ($P < 0{\cdot}01$). There were more deaths from cancer of the colon and fewer from cancer of the oesophagus than expected, but the differences are not significant.

*Mortality by socio-economic group*

Some cancers vary in incidence according to socio-economic group. In England and Wales death from cancer of the colon was slightly more common in socio-economic Group 1, that is professional occupations (standardized mortality ratio (SMR) 104, ages 15–64, proportioned

TABLE IV.—*Malignant neoplasm of the digestive tract*

| | Actual deaths in brewery workers 1954–63 | Expected deaths in brewery workers: based on All Ireland death rates 1958–60 | Expected deaths in brewery workers: based on Dublin County Borough death rates 1958–60 | Actual deaths in brewery workers 1964–73 | Expected deaths in brewery workers: based on All Ireland death rates 1968–60* | Expected deaths in brewery workers: based on Dublin County Borough death rates 1969, 1971–75 |
|---|---|---|---|---|---|---|
| 19a Malignant neoplasm of stomach | 24 | 22·4 | 26·2 | 16 | 16·0 | 16·2 |
| 19b Malignant neoplasm of other digestive organs and peritoneum Codes 150, 152–159 | *54* | *32·8* | *45·8** | *47* | *29·0* | *40·3*** |
| 150—Oesophagus | 5 | 4·2 | 8·9 | 5 | 3·8 | 6·9 |
| 152—Small intestine, incl. duodenum | 1 | 0·5 | 0·7 | — | 0·2 | 0·5 |
| 153—Large intestine, except rectum | 16 | 11·4 | 14·7 | 16 | 10·4 | 12·6 |
| 154—Rectum and rectosigmoid junction | 17 | 7·5 | 9·8 | 15 | 6·1 | 8·4 |
| 155—Liver and intrahepatic bile duct | 3 | 1·0 | 3·0 | 4 | 1·5 | 2·5 |
| 156—Gall bladder and bile ducts | 1 | 2·6 | 1·0 | 1 | 0·7 | 1·1 |
| 157—Pancreas | 11 | 5·1 | 6·5 | 6 | 6·0 | 7·5 |
| 158—Peritoneum and retroperitoneal tissue | — | 0·4 | — | — | 0·1 | 0·3 |
| 159—Unspecified digestive organs | — | 0·1 | 0·8 | — | 0·2 | 0·5 |

* Deaths found Dublin County Borough males 1958–60—352 (expected 45·4). Deaths registered Dublin County Borough males 1958–60—357 (expected 45·8).
** 1969, 1971–75 incl.

TABLE V.—*Actual and expected deaths for brewery blue-collar employees for certain causes adjusted for socio-economic group*

| Cause of death | Actual deaths in brewery workers 1954–73 | Expected deaths in brewery workers based on Dublin County Borough all socio-economic groups 1954–73 | SMR for skilled and unskilled manual workers (group 8-X). All Dublin males (1971–74)* | Expected deaths in brewery workers based on SMR for Dublin skilled and unskilled manual workers, (1971–74) |
|---|---|---|---|---|
| Ca colon | 32 | 27·3 | 88·1 | 24·1 |
| Ca rectum | 32 | 18·2 | 108·0 | 19·7 |
| Ca oesophagus | 10 | 15·8 | 92·4 | 14·6 |
| Ca stomach | 40 | 42·4 | 116·1 | 49·2 |
| Ca pancreas | 17 | 14·0 | 108·9 | 15·2 |
| Ca lung | 98 | 99·2 | 109·1 | 108·2 |
| Cirrhosis liver | 11 | 7·6 | 87·1 | 6·6 |
| Bronchitis | 128 | 154·9 | 120·1 | 186·0 |
| Ischaemic heart disease | 430 | 470·5 | 97·1 | 456·7 |

* Based on unpublished figures and approximate only, using 1971 census and deaths in Dublin males 1971–1974.

mortality ratio (PMR) 138, ages 65–74). (1969–73). Cancer of the rectum, on the other hand, was slightly more common among Group V, unskilled occupations (SMR 108, ages 15–64, PMR 94, ages 65–74), but not more common among Group IV, partly skilled occupations, and is less common in Group I (SMR 84, ages 15–64, PMR 101, ages 65–74). In England and Wales cancer of the colon is slightly more common, therefore, in the upper socio-economic group and cancer of the rectum in the lowest socio-economic group (Registrar General's Decennial Supplement, England & Wales, 1971).

In Ireland, no study of mortality by socio-economic group has previously been undertaken. For the purpose of this research the Central Statistics Office, Dublin agreed to analyse certain causes of death in Dublin males for the years 1971–74, inclusive, by age group and socio-economic group. A similar analysis was made of the Dublin male population at the 1971 census. The Irish socio-economic group changes are tentative because of the large group coded "unclassified". However, the pattern is similar to that in England and Wales.

When the expected number of deaths for cancer of the rectum among the brewery workers is adjusted, using the SMR for the group "skilled and unskilled manual workers" (Group 8-X) for Dublin males (1971–74), the expected number increases from 18·2 to 19·7 (Table V). If "other non-manual workers" (Group 7) are included the expected number is reduced to 16·4. The brewery blue-collar workers undoubtedly include some who would be classified as "other manual workers".

Cancer of the colon, similarly adjusted, has an expected number of deaths for skilled and unskilled manual workers of 24·1. The difference with the actual deaths, 32, is not significant. There were fewer than the expected deaths among the brewery workers adjusted for socio-economic group for cancer of the oesophagus, stomach and lung, but the differences were not significant.

Both in England and Wales and in Ireland the adjustments necessary to correct for socio-economic group differences in cancer of the rectum and colon are small. When these adjustments have been made, there is still a significantly higher than expected number of deaths from cancer of the rectum among the brewery employees ($P < 0{\cdot}01$).

*Place of work in the brewery*

We wondered whether an excess of those who died of cancer of the rectum had worked in any one department within the brewery. In order to select suitable controls, those who died from cancer of the rectum were matched with 3 brewery workers who died of other causes. These controls were the next 3 men who died who were within the same 5-year age group at age of death as the cancer of the rectum death. When analysed, the cancer and control group differed significantly with regard to place of work in the brewery. In order to confirm that the control group was representative of the distribution of workers within the brewery, a further 3 controls were chosen, in a method similar to that oultined above, for each rectal cancer death. This gave a total of 6 controls per rectal cancer death.

The length of service in the brewery, the departments in which they worked, and the number of years spent in each department were found from the work records. This information was available for 30/32 cancer of the rectum deaths, 30/32 cancer of the colon deaths and 172/192 controls.

The mean length of service in the brewery for the 3 groups of workers was:

| | |
|---|---|
| Cancer of the rectum: | 36·1 years |
| Cancer of the colon: | 34·7 years |
| Controls: | 35·2 years |

The men worked in the following 6 areas in the brewery: engineering department, brewhouse, cooperage, traffic, administration and "other departments" (Table VI).

TABLE VI.—*A Dublin brewery. Number of men and number of years worked in departments tabulated by cause of death*

| | Ca rectum | | Ca colon | | Controls (other causes) | |
|---|---|---|---|---|---|---|
| Department | Men | Years | Men | Years | Men | Years |
| Brewhouse | 12 | 300·4 | 8 | 181·7 | 35 | 1099·4 |
| Engineers | 8 | 286·3 | 9 | 234·7 | 68 | 2134·7 |
| Cooperage | 7 | 228·8 | 9 | 248·6 | 50 | 1241·7 |
| Traffic | 5 | 110·1 | 8 | 291·8 | 41 | 1155·5 |
| Admin. | 4 | 68·9 | 3 | 37·2 | 3 | 27·4 |
| Others | 3 | 87·1 | 2 | 44·5 | 19 | 323·3 |
| Total | 30 | 1081·6 | 30 | 1039·5 | 172 | 5982·1 |

Some of the men worked in more than one department. There was no significant difference between the proportion of men with cancer of the rectum and the proportion of the control group who worked in the engineering department, cooperage, traffic or in "other departments". Significantly more of the men who died of cancer of the rectum worked in administration than in the control group. However, the number of years spent in administration by either group was small. Of the 30 men who died of cancer of the rectum, and for whom place of work was known, 12 men (40%) worked in the brewhouse. Out of 172 controls for whom place of work was known 35 (20·3%) worked in the brewhouse. Significantly more of those who died of cancer of the rectum worked in the brewhouse than in the control group ($\chi^2$ with Yates' correction = 4·48, d.f. = 1; $0{\cdot}05 > P > 0{\cdot}025$).

The expected number of deaths from cancer of the rectum among the brewery blue-collar workers was 18·2, based on death rates for males in Dublin County Borough. The distribution of these deaths within the brewery would be expected to be in proportion to the number of men working in each department. Thus 35/172 controls (20·3%) worked in the brewhouse, so 20·3% of the 18·2 expected deaths, or 3·7, would have been expected to occur in those who worked there. In fact 12 of those who died of cancer of the rectum were known to have worked in the brewhouse. The observed number of deaths was significantly greater than the number expected among those who worked in the brewhouse ($\chi^2 = 18{\cdot}62$, $P < 0{\cdot}001$). 14·5 deaths would have been expected among those who worked in areas other than the brewhouse. Eighteen of those dying of cancer of the rectum did not work in the brewhouse, and there were 2 men for whom place of work was not known. Those who worked outside the brewhouse had a greater than expected number of deaths from cancer of the rectum, but this increase was not significant ($\chi^2 = 2{\cdot}09$).

*Drinking pattern of deceased described by relatives*

The relatives of those who died of cancer of the rectum were sought and were questioned with regard to the deceased's leisure-time activities, which included their drinking habits. For each relative traced, 2 control relatives were sought from among men who had died of other causes in the same age group, matched as closely as possible for age at death and the year in which they died. It was possible to trace the relatives of 16/32 who had died of cancer of the rectum, among whom 15 drank stout, and 29 control relatives, among whom 27 drank stout.

The mean alcohol intake of those who died of cancer of the rectum was reported by the next-of-kin to have been 30·9 pints (17·6 litres) of stout per week and 1·8 glasses of spirits per week (1 glass = 71 ml). The stout intake reported was inflated by one informant with a high intake. Without this case the mean intake of stout is reduced to 23·6 pints (13·4 litres) of stout per week. The mean intake for the 29 controls was 16·1 pints (9·1 litres) of stout per week and 4 glasses of spirits per week. Excluding the man with the reported high intake there is still a significant difference between the reported stout consumption of the remaining 15 rectal cancer patients and the 29 controls ($t$ test, 42 d.f., $0{\cdot}05 > P > 0{\cdot}02$).

The difference in the reported drinking patterns can be contrasted with the reported smoking pattern of the same persons. Nine out of the 16 relatives of the rectal cancer deaths reported that the deceased smoked cigarettes, and the mean number of cigarettes smoked by the smokers was 24/day, or a mean of 13·5/day for all 16. Seventeen of the 29 controls smoked cigarettes, and the mean number of cigarettes smoked by the smokers was 22·3/day, or a mean of 13·1 for all 29, showing no significant difference in smoking pattern between the 2 groups.

*Urban/rural gradients in beer consumption and mortality*

The greatest consumption of stout in the

TABLE VII.—*Expected number of deaths for brewery workers* 1964–73 *if they had had the same risk as the populations of Dublin County Borough, other urban and rural areas,* 1969, 1971–75 *inclusive. Populations based on* 1971 *census*

| | Actual brewery deaths 1964–73 incl. | Expected number of brewery deaths 1964–73 based on death rates in: | | | |
|---|---|---|---|---|---|
| | | All Ireland | Dublin County Borough | Other urban areas | Rural areas |
| B19b, Codes 150, 152–159 incl. Malignant noeplasm of other digestive organs and peritoneum: | *47* | *31·2* | *40·3* | *35·9* | *28·4* |
| 150—Oesophagus | 5 | 4·1 | 6·9 | 4·8 | 3·4 |
| 152—Small intestine, incl. duodenum | — | 0·3 | 0·5 | 0·5 | 0·3 |
| 153—Large intestine, except rectum | 16 | 11·2 | 12·6 | 13·9 | 10·2 |
| 154—Rectum and rectosigmoid junction | 15 | 6·5 | 8·4 | 8·1 | 5·7 |
| 155—Liver and intrahepatic bile ducts | 4 | 1·7 | 2·5 | 1·4 | 1·7 |
| 156—Gall bladder and bile ducts | 1 | 0·7 | 1·1 | 0·8 | 0·7 |
| 157—Pancreas | 6 | 6·2 | 7·5 | 5·9 | 6·0 |
| 158—Peritoneum and retroperitoneal tissue | — | 0·3 | 0·3 | 0·4 | 0·2 |
| 157—Unspecified digestive organs | — | 0·2 | 0·5 | 0·1 | 0·2 |

general population is in men between the ages of 25 and 44 years among the blue-collar workers (social class coded C2 and DE) and there is a higher consumption in Dublin County Borough than in the large towns, with the lowest consumption in the rural areas (Public Attitudes Surveys, 1975). The urban/rural difference for all causes of death in the Republic of Ireland has been reported elsewhere (Ward *et al.*, 1977).

The expected number of deaths among the brewery workers if they had the same risk as males in Dublin County Borough, other urban areas and rural areas of the Republic of Ireland for malignant neoplasms of other digestive organs and peritoneum (B19b) is shown in Table VII. The risk for cancer of the oesophagus, rectum, liver and gall bladder is highest in the Dublin County Borough, and higher in other urban areas than in rural areas.

There is a gradient, therefore, for both stout consumption and death risk from cancer of the rectum which is high among the brewery blue-collar workers, less among Dublin County Borough males (especially among skilled and unskilled manual workers), less still in other urban areas, and the lowest in the rural areas.

### DISCUSSION

This study was undertaken to see whether there was an association between a high consumption of beer and cancer of the rectum and colon. By tradition, the brewery blue-collar workers are high consumers of beer, usually in the form of stout, and have in the past had easy access to beer at the brewery. The high consumption was confirmed by the study in Boston and Ireland (Brown *et al.*, 1970).

The workers at the Dublin brewery are carefully chosen and are provided with excellent social services, security of tenure, a good pension and good medical care throughout service and retirement. Smoking was forbidden at the brewery for many years, which may account for the lower death rate from bronchitis, pneumonia and perhaps some other conditions. The Dublin brewery workers differ from workers in other breweries in that they generally join the brewery in their late teens and stay with the brewery for life. The blue-collar workers all belong to the staff pension scheme, and careful records are kept of all deaths among the employees and pensioners. Therefore, no deaths are likely to be overlooked. Stout is the traditional drink of the Dublin working man, and probably much less spirits are consumed by the Dublin brewery workers than might be the case by workers in breweries elsewhere, who perhaps join a brewery for only part of their working life.

The brewery workers tend to be overweight, and death certified as from diabetes is more common among them than among the general Dublin male population. Nevertheless, their average expectation of life was 70·7 years in 1954–63 and 72·3 years in 1964–73, and their pattern of death is not that expected of a population consuming large amounts of alcohol. This may be because, while they consume, on the whole, above-average amounts of stout and other beers, they do not drink much spirits and they maintain their general nutrition and do not have the gastritis and lack of appetite that occurs with spirit drinkers. They do not have an increased death rate from cancer of the oesophagus, pharynx or liver, from accidents or suicide, or a significantly increased death rate from cirrhosis of the liver.

One cancer is significantly more common among the Dublin brewery blue-collar workers: cancer of the rectum (32 with 18·2 expected, Table V). Nevertheless, cancer of the rectum accounts for less than 2% of the deaths. Cancer of the colon is also more common (32 with 27·3 expected) but the difference is not statistically significant. A higher death rate from cancer of the rectum among workers at a brewery, especially those working in the brewhouse, does not prove that drinking beer causes this cancer. It could be that there is some unknown factor associated with working in the brewhouse which increases the risk of cancer of the large bowel. The findings of this study require comparison with studies in other breweries, and also with distillery workers who will be more inclined to drink spirits, especially if it is provided by the distillery.

Reduced consumption of fibre in the diet has been associated with this form of cancer (International Agency for Research on Cancer, 1977). Those who consume a large amount of stout with its high calorie value, may reduce their fibre intake. It has been noted that some of those at the brewery who gave up drinking for Lent, the period of abstinence before Easter, are troubled by constipation, and stout and other beers appear to hasten rather than retard the evacuation of the bowel.

The increased death rate from cancer of the rectum among the Dublin brewery blue-collar workers and pensioners, and the gradient for both stout consumption and for deaths from cancer of the rectum among the different male socio-economic and place-of-residence groups in Ireland, does suggest a relationship between high consumption of beer and cancer of the bowel.

Cancer of the rectum in men is more common in England than in the Republic of Ireland (Institute of Cancer Research, 1976*a*, *b*). Until 1970 the consumption of beer was higher in England than in Ireland (Produktschap voor Gedistilleerde Dranken, 1977). On the other hand, much more stout has always been drunk in Ireland than in England. Further research into the past drinking behaviour of patients with cancer of the rectum and colon and of controls is required. The possibility of a carcinogenic substance in the stool of those who had a high consumption of different forms of beer should be investigated.

It is important to emphasize that the expectation of life of the Dublin brewery blue-collar workers and pensioners is good and is slightly better than the average for males of all social classes living in Dublin.

We would like to thank the Directors of the brewery concerned and their medical department for agreeing to assist us with this study. The staff in the personnel division gave us great assistance by allowing us access to their records and providing lists of the brewery employees and pensioners who had died during the 20-year period of the study.

Our thanks are due to the Minister for Health, to An t'Ard-Chláraitheoir (the Register General) and to Mr M. Barrett and his staff at the General Register Office, Dublin for their assistance in providing us with copies of the death certificates. We also thank the staffs of the General Register Offices in London, Edinburgh and Belfast for their help in tracing the death certificates of those who died outside the Republic of Ireland. We were assisted in tracing the death certificates by Miss C. Stafford and Mrs T. Mullan.

We are very grateful to Mr T. Linehan, the Director, and Mr W. Keating and Miss M. G. Nugent of the Central Statistics Office, Dublin, for their help in providing statistics of male deaths in Dublin and for coding the death certificates, and to Dr S. P. H.

Mandel, consultant to the International Agency for Research on Cancer, Lyon for statistical advice.

This study was supported by the International Agency for Research on Cancer, Lyon and by the National Institute for Alcohol Abuse and Alcoholism, Bethesda, Maryland, U.S.A.

### REFERENCES

Breslow, N. E. & Enstrom, J. E. (1974) Geographic correlations between cancer mortality rates and alcohol–tobacco consumption in the United States. *J. Natl Cancer Inst.*, **53**, 631.

Brown, J., Bourke, G. F., Gearty, G. F. & 16 others (1970) Nutritional and epidemiologic factors related to heart disease. *World Review of Nutrition and Dietetics*, **12**, 1. Basel: Karger.

International Agency for Research on Cancer, Intestinal Microecology Group (1977) Dietary fibre, transit-time, faecal bacteria, steroids and colon cancer in two Scandinavian populations. *Lancet*, ii, 207.

Institute of Cancer Research (1976*a*) *Serial Mortality Tables Neoplastic Diseases, Vol.* 1. *England and Wales*, 1911–70. London.

Institute of Cancer Research (1976*b*) *Serial Mortality Tables Neoplastic Diseases, Vol.* 2. *Ireland* (*Republic*), 1922–70. London.

Produktschap voor Gedistilleerde Dranken (1977) *Hoeveel alcoholhoudende dranken warden er in de wereld gedronken?* Nederland: Schiedam.

Public Attitudes Surveys Ltd (1975) *Drinking Behaviour in the Republic of Ireland*, 1974/75. England.

Registrar General's Decennial Supplement, England & Wales (1971) *Occupational Mortality Tables*. London: HMSO.

Ward, J., Healy, C. & Dean, G. (1977) Urban and rural mortality in the Republic of Ireland. *J. Irish Med. Assoc.*, **71**, 73.